# Sonication dissociates the synaptic cleft and allows purification of postsynaptic densities with associated postsynaptic membrane

**DOI:** 10.1186/s13041-025-01217-7

**Published:** 2025-05-30

**Authors:** Ayse Dosemeci, Jung-Hwa Tao-Cheng

**Affiliations:** 1 Structural Cell Biology Section, National Institutes of Health, Bethesda, MD 20892 USA; 2 NINDS Electron Microscopy Facility National Institute of Neurological Disorders and Stroke, National Institutes of Health, Bethesda, MD 20892 USA

**Keywords:** PSD, Electron microscopy, EM, Synaptic junction, Synaptosome

## Abstract

In the synaptic junction, pre-and post compartments are anchored to each other through trans-synaptic bridges spanning the synaptic cleft. Here we demonstrate that mild mechanical disturbance through sonication dissociates the synaptic cleft, and releases PSDs adjoined to the postsynaptic membrane, but devoid of presynaptic elements. It is the first time, to our knowledge, that dissection of the synaptic cleft has been achieved without the use of chemical/enzymatic treatments. This observation suggests that some of the protein-protein interactions involved in the anchoring of pre- and postsynaptic compartments are relatively weak non-covalent associations. We describe a method for the further fractionation of PSDs with the associated postsynaptic membrane. This PSD preparation provides a valuable tool for studies of postsynaptic membrane components, such as glutamatergic receptors, in an environment closer to their native state.

**Supplementary Information:**

The online version contains supplementary material available at 10.1186/s13041-025-01217-7.

## Introduction

The synaptic cleft, a ~ 20 nm wide uniform space, separates pre- and post-synaptic membranes [1]. Multiple trans-synaptic bridges that maintain this close apposition appear to remain stable under various cell fractionation strategies. Thus, sub-cellular elements with intact synaptic junctions such as synaptosomes (presynaptic sacs with attached PSDs) can be isolated by serial centrifugal fractionation of homogenates from brain tissue.

Further fractionation of synaptosomal preparations to separate the pre- and postsynaptic elements requires dissociation of the cleft. Postsynaptic densities devoid of presynaptic elements as well as of junctional membranes are conventionally obtained by treatment of synaptosomes with detergents [2, 3], which promote solubilization of presynaptic components dependent on pH [4]. Another study [5] used proteolytic enzymes to break down transsynaptic bridges and separate pre- and postsynaptic compartments, a strategy expected to retain junctional membranes but likely to cause breakdown of exposed PSD proteins as well.

PSD preparations, obtained by treatment of synaptosomal fractions with the detergent Triton-X100, have been widely used for biochemical [Reviews: [6, 7] and morphological [8–13] analysis of the PSD complex by a variety of proteomic and electron microscopy (EM) techniques. Although studies with Triton-derived PSDs yielded a wealth of information on the molecular composition and architecture of the PSD, a major concern has been possible detergent-induced alterations in the PSD structure and in protein-protein interactions. Notably, the complete removal of the postsynaptic membrane is expected to affect those membrane-bound PSD elements. Thus, to study the structure, orientation and interactions of PSD elements such as neurotransmitter receptors, a preparation that maintains postsynaptic membrane integrity would be desirable.

Attempts to isolate PSDs while preserving the adjoining postsynaptic membrane have been hampered by the stability of transsynaptic bridges. Although certain transsynaptic bridges such as N-cadherins are known to be Ca^2+^-dependent, exposure of hippocampal neurons to EGTA promotes only partial opening of synaptic clefts in a minority (17%) of synapses [14], suggesting the existence of additional, calcium-independent trans-synaptic bridges.

In the present study we aimed to design a strategy for isolating PSDs without the use of detergents in order to preserve the adjoining postsynaptic membrane. We find that mechanical disturbance through sonication dissects the synaptic cleft to yield intact looking PSDs with attached postsynaptic membranes. We further purify and analyze this PSD fraction to verify morphological purity and the presence of major PSD scaffolds and glutamate receptors.

## Methods

### Antibodies used

(Protein: Company (Catalogue #) host/type dilution for Western)

PSD-95: New England Peptide (custom) rabbit polyclonal 1:1000;

SynGAPα2: Abcam (ab77235) rabbit monoclonal (EPR2883Y) 1:1000;

Homer1: Synaptic Systems (160 011) mouse monoclonal (2G8) 1:500;

SNAP25: Chemicon (MAB331) mouse monoclonal (SP14)1:500;

Syntaxin1: Chemicon (MAB336) mouse monoclonal (SP8)1:500;

GluA2: Millipore (MAB397) mouse monoclonal (6C4)1:500;

GluN2A/B: Millipore (AB1548) rabbit polyclonal 1:100.

### Preparation of synaptosomal fractions

Brains from 7 to 12 week-old rats of both sexes were custom collected by Rockland (Gilbertsville, PA). Briefly, brains were collected within two minutes after cervical dislocation and were frozen in liquid nitrogen. Frozen brains were kept at -80° C until use. Brains were thawed for 1 min by immersion in 0.32 M sucrose at 37° C. Cerebral cortices were dissected and immediately homogenized in 0.32 M sucrose. The homogenate was centrifuged (900 g for 10 min) to pellet nuclei. The supernatant (S1) fraction was centrifuged at 10,500 g for 12 min. Resulting pellet (P2) was resuspended in isotonic sucrose and fractionated on a sucrose gradient to separate mitochondria (pellet through 1.2 M sucrose) and synaptosomes (1/1.2 M sucrose interphase). Synaptic plasma membrane (SPM) fractions were obtained by suspension of synaptosomes in hypotonic medium (1 mM HEPES) and centrifugation (10,500 g for 15 min). Synaptosome and SPM fractions were stored at -20˚ C in 33% glycerol until use.

### Sonication

A probe sonicator, Kontes KT50 micro ultrasonic cell disruptor (rated power output 50 W, frequency 20 kHz), was used for sonication. Synaptosomal fractions were resuspended into 3 ml of 20 mM HEPES pH 7 and were sonicated at an output setting of 40%, in a tube placed on ice, four times for 20 s each, with 40 s cooling intervals. When indicated, a parallel unsonicated control sample was set aside in ice.

### Purification of PSDs with associated postsynaptic membrane

SPM fractions containing 3.6 mg protein were resuspended into 3 ml with 20 mM HEPES and sonicated as described above. Samples were layered on top of a gradient consisting of 1.2 M (3 ml) and 0.85 M (6 ml) sucrose layers and centrifuged using a Beckman SW41 rotor at 40,000 rpm for 90 min. The pellets through 1.2 M sucrose were collected. For EM analysis, pellets were resuspended into 1 mM HEPES and re-pelleted on a bench centrifuge before fixation.

### Electrophoresis and western immunoblotting

Protein in supernatants was recovered by precipitation with TCA and re-solubilized in SDS-containing sample buffer before electrophoresis. 4–15% Mini-PROTEAN TGX Precast polyacrylamide gels (BioRad) were used for SDS-PAGE. Gels were transferred to PVDF membranes using the Trans-Blot Turbo Transfer System (BioRad), blocked, incubated with primary and secondary antibodies, and visualized via chemiluminescence.

### Electron microscopy

Pellets were fixed with 4% glutaraldehyde in 0.1 M cacodylate buffer at pH 7.4 at room temperature and stored at 4˚ C. Fixed pellets were processed in the centrifuge tubes until the embedding step. Samples were washed in 0.1 M cacodylate buffer and treated with 1% osmium tetroxide in 0.1 M cacodylate buffer for 1 h on ice, washed and treated with 1% uranyl acetate in acetate buffer at pH 5.0 at 4˚ C overnight. Samples were then washed and dehydrated through a graded series of ethanol and embedded in epoxy resins. Prior to embedding, pellets were removed from the centrifuge tubes, trimmed and oriented so that they were sectioned vertically through the thickest portion of the pellets. Sections of 70–90 nm thickness were counterstained with lead citrate. Images were photographed with a bottom-mounted digital CCD camera (AMT XR-100, Danvers, MA, USA) on a JEOL 1200 EX transmission electron microscope. In order to document the gradient of components within the pellets, photographs were taken consecutively from top to bottom of the pellets at 10,000 X. Images for showing details of the prep were taken at 40,000 X magnification.

## Results

Synaptosome fractions were sonicated as described in methods. Sonicated and parallel unsonicated ‘control’ samples were layered on sucrose gradients (1 ml each of 0.32 M and 0.85 M sucrose) and centrifuged (50,000 rpm for 1 h in a Beckmann SW55 rotor) to remove soluble and light particulate material. The denser material pelleted through 0.85 M sucrose was collected and compacted through centrifugation in 20 mM HEPES and fixed for EM analysis.

**Fig. 1:**
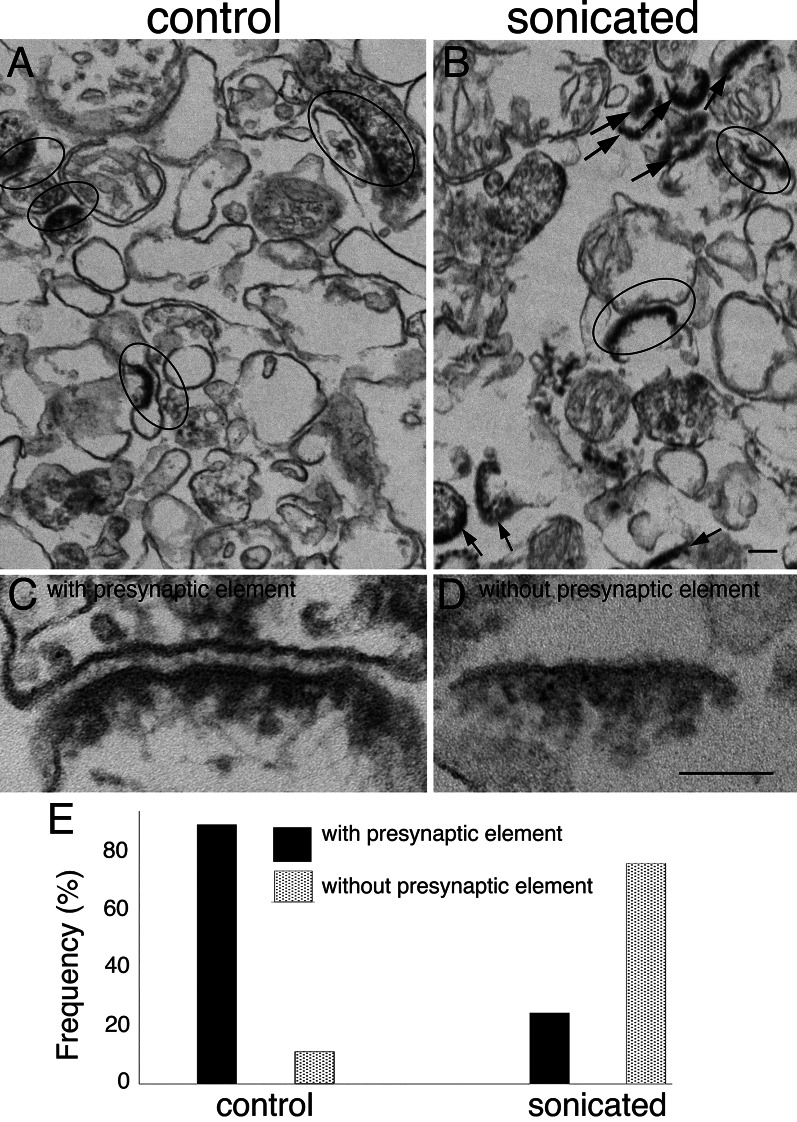
Effect of sonication on synaptic junctions. Synaptosome preparation (300 µg protein in 3 ml) is sonicated, pelleted through 0.85% sucrose, fixed and examined by electron microscopy. Unsonicated control samples (**A** & **C**) mostly exhibit intact synaptic junctions with apposed pre- and postsynaptic membranes (encircled), whereas in sonicated samples (**B** & **D**) PSDs without adjoining presynaptic elements (arrows in B) are predominant. PSDs in sonicated samples have associated postsynaptic membranes (**D**). Scale bars = 100 nm. Quantitative evaluation of data from one experiment, where parallel samples were scored for PSD-containing structures (control *n* = 80; sonicated *n* = 130) shows drastic increase in PSDs without presynaptic elements upon sonication (**E**)

EM examination showed PSD-containing structures in both control and sonicated pellets, either with or without presynaptic attachments (Fig. 1). In order to assess the relative number of clefts dissociated upon sonication, each PSD-containing structure was classified as either a PSD ‘with presynaptic elements’ (Fig. 1C) or a bare PSD ‘without pre-synaptic elements’ (Fig. 1D). Control pellets mostly exhibited intact synaptic junctions with PSDs apposed to presynaptic elements (89%, Fig. 1E). In sonicated pellets, on the other hand, only one in four of PSDs were with presynaptic elements (Fig. 1E).

Closer examination of the PSDs devoid of presynaptic elements in sonicated pellets revealed that they still retained the adjoining postsynaptic membrane (Fig. 1D). In an attempt to devise a strategy for the further purification of these structures, we reasoned that PSDs without the presynaptic elements (Fig. 1D) would have a lower membrane to protein ratio compared to intact junctions with both pre- and postsynaptic membranes (Fig. 1C) and therefore should be of higher density. Thus, a density-based fractionation protocol was implemented for the separation of PSDs from intact synaptic junctions.

SPM preparations (pellets from lysed synaptosomes) were sonicated and fractionated by density-gradient centrifugation in a discontinuous gradient consisting of 0.85 M and 1.2 M sucrose layers as described in Methods. Since synaptosomes and SPM fractions containing synaptic junctions are conventionally collected at the interphase above the 1.2 M sucrose, it was predicted that material with intact junctions would be retained at this interphase while the denser PSDs would pellet through. Lighter particulate material, on the other hand, is expected to be retained at the interphase above 0.85 M sucrose, while soluble proteins would remain in the supernatant in 20 mM HEPES buffer.

**Fig. 2:**
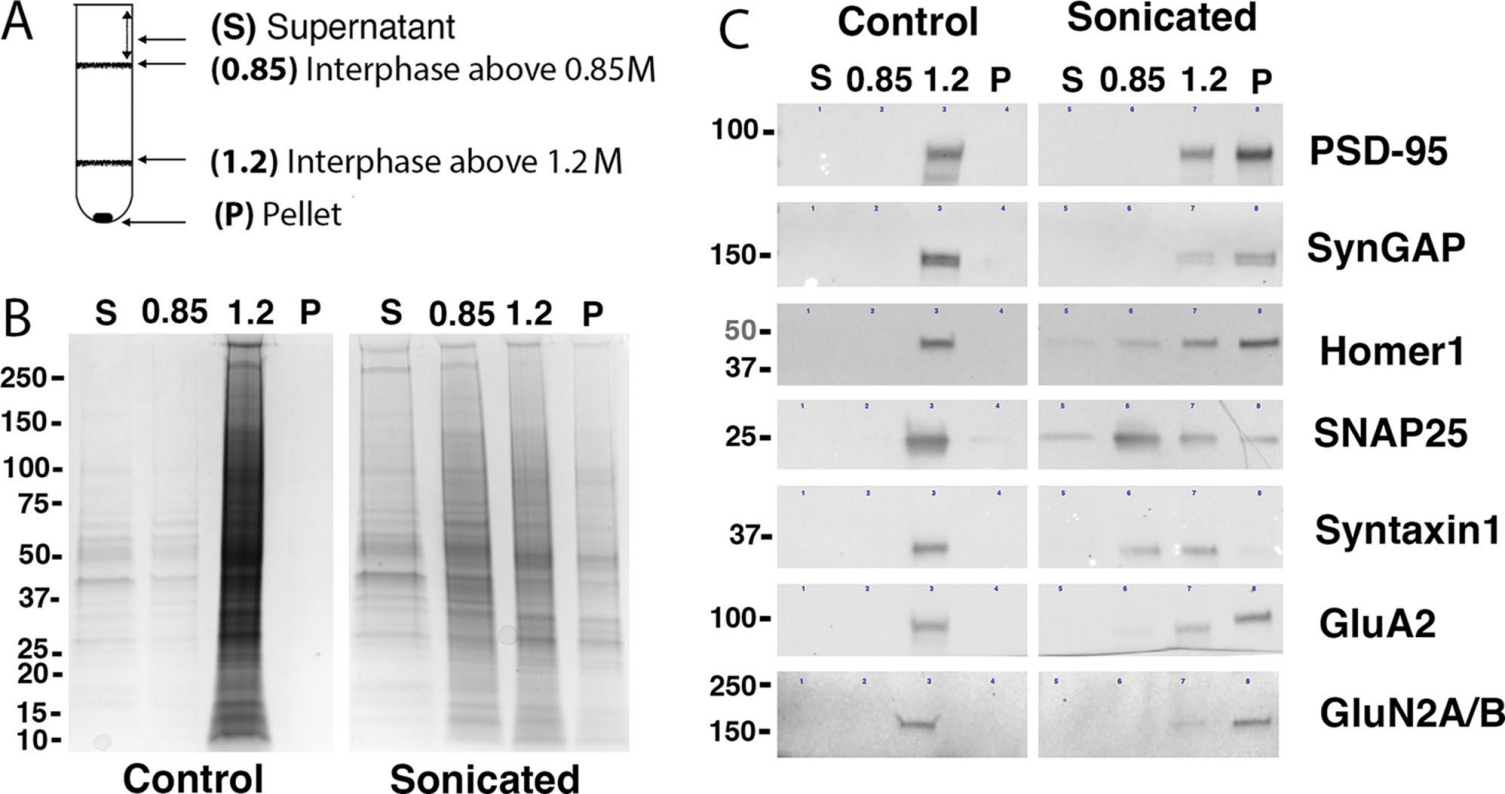
Purification of PSDs with associated postsynaptic membrane: fractionation of sonicated samples. Control and sonicated synaptic plasma membrane (SPM) preparations are fractionated by centrifugation through a sucrose gradient. Material is collected from four fractions: supernatant, interphases over 0.85 M and 1.2 M sucrose layers and pellet (**A**). Fractions corresponding to control and sonicated samples are analyzed by PAGE, Coomassie Blue staining (**B**) and Western Immunoblotting (**C**). Each lane in B and C corresponds to material recovered in indicated fraction from the same starting amount of SPM preparation

Pelleted material as well as material from the two interphases and the supernatant were collected (Fig. 2A) and analyzed for protein content. Figure 2B shows Coomassie stains of fractions recovered from the same amount of starting material. As expected, virtually all proteins from un-sonicated control samples were recovered at the interphase above 1.2 M sucrose layer, indicating preservation of the synaptic junctions. In contrast, proteins from sonicated samples were distributed throughout the gradient, reflecting breakdown of synaptic junctions (Fig. 2B).

Immunoblots in Fig. 2C show the fractionation of specific synaptic proteins upon sonication. Again, lanes represent fractions recovered from the same amount of starting material. In accordance with the Coomassie protein profile, in control samples, all of the proteins tested were basically recovered at the 1.2 M interphase. On the other hand, in sonicated samples, the proteins became distributed among two or more fractions.

Upon sonication, major PSD scaffolds, PSD-95, SynGAP and Homer1, as well as components of AMPA and NMDA types of glutamate receptors, GluR1 and NR2A/B became most prominent in pellets (Fig. 2C, Supplementary Table 1). Considering that the pellet represents a much smaller proportion of the total protein recovered (Fig. 2B), the results indicate a high degree of enrichment of PSD-specific proteins [15] in that fraction.

On the other hand, presynaptic components [16], syntaxin, an integral membrane protein, and SNAP25, a membrane-associated presynaptic protein, fractionated predominantly into 0.85 and 1.2 M sucrose interphases, with relatively low levels in pellets. SNAP25 showed a wider distribution, suggesting some dissociation and redistribution during sonication. All of the proteins tested were also detected at the 1.2 M interphase, but at reduced levels, presumably corresponding to the minor pool of synaptic junctions that remain intact after sonication.

**Fig. 3:**
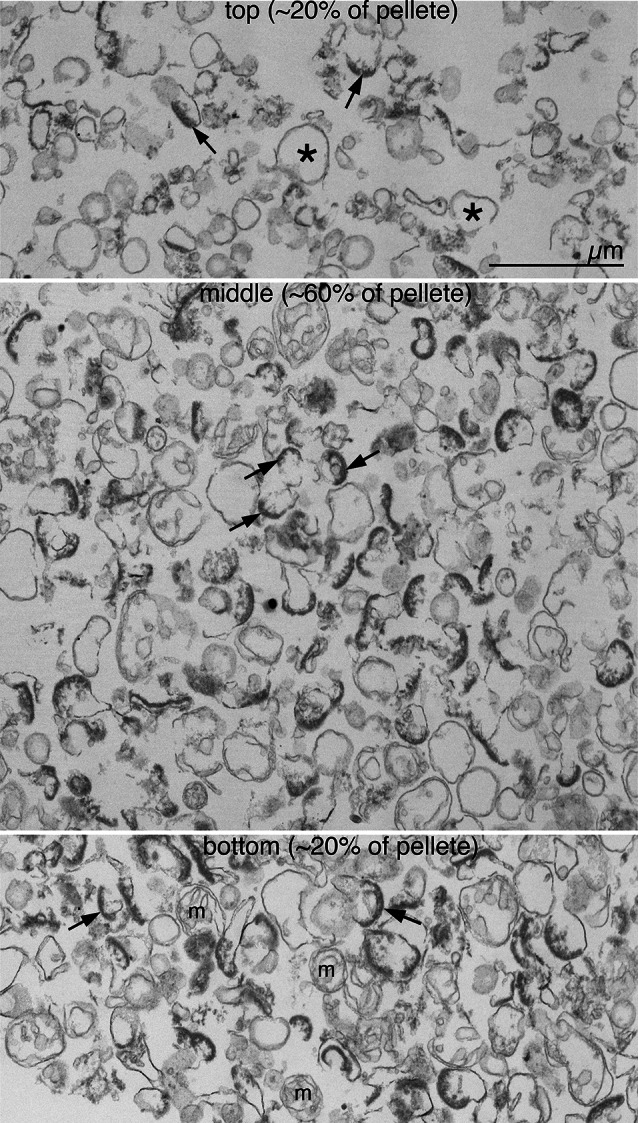
Purification of PSDs with associated postsynaptic membrane. Examination of pellets from sonicated samples by electron microscopy reveals predominance of PSDs (arrows) with associated postsynaptic membranes but without presynaptic elements. Contaminants include membranous elements (asterisks) which are more prevalent in the top of the pellet, and mitochondria (m) which are more abundant in the bottom of the pellet. Scale bar = 1 μm

EM observation of glutaraldehyde-fixed pellets from sonicated samples (Fig. 3) showed PSDs with associated postsynaptic membranes from the top to the bottom of the cross-sectioned pellet. The high yield of PSDs without presynaptic elements upon sonication was verified in three preparations, with an average of 96 ± 1% of PSD-containing profiles without presynaptic elements [two experiments using standard SPM preparations: 95.5% (173/181), 93.4% (113/121), and one using an SPM preparation from older (24–48 weeks) rats: 96.6% (256/265)].

**Fig. 4:**
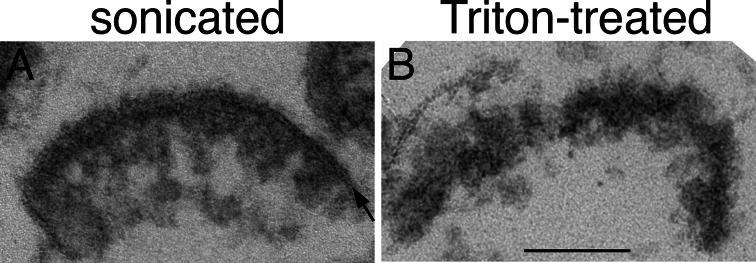
PSDs separated by sonication (**A**) exhibit an associated postsynaptic membrane (arrow), whereas PSDs in the classical Triton-derived preparation (**B**) are devoid of postsynaptic membrane. Scale bar = 100 nm

The most widely used protocol for the preparation of PSD-enriched fractions, devised by Cohen et al., [2] involves treatment of synaptosomal fractions with TritonX-100. Figure 4 allows a comparison of Triton-derived PSDs and sonication-derived PSDs from the present study. While PSDs appear to conserve their in situ morphology under either protocol, only PSDs separated through sonication retain the adjoining postsynaptic membrane (arrow in Fig. 4).

## Discussion

In the present study we demonstrate that mechanical disturbance of synaptosomal fractions through sonication causes dissociation of the synaptic junction to separate PSDs with adjoining postsynaptic membrane. Examination of the sonicated fractions by EM reveals that a majority (75%) of synaptic junctions had been dissociated, with the release of PSDs devoid of presynaptic attachment.

Dissociation of the synaptic junction upon sonication indicates that some of the protein-protein interactions that are responsible for the adherence of pre- and postsynaptic compartments are relatively weak bonds, susceptible to mechanical disturbance. The susceptible bonds, likely non-covalent associations, may either be those between pre- and postsynaptic adhesion partners, or those between adhesion molecules and their anchoring elements at either pre- or postsynaptic compartments. Future studies could identify these weak associations and examine their potential role in the disruption of synaptic contacts in situ.

To our knowledge, it is the first time, that dissection of the synaptic cleft has been achieved by mechanical disruption, without the use of chemical or enzymatic treatments. In addition to its potential relevance for the clarification of nature of association, demonstration of the dissociation of synaptic junctions by sonication, opens the way for a new strategy for the purification of PSDs.

We took advantage of the expected density difference to separate dissociated PSDs from remaining intact synaptic junctions. Biochemical analyses of fractions, separated by density gradient centrifugation show high enrichment of known PSD components as well as glutamate receptors in pellets through 1.2 M sucrose, whereas presynaptic elements are segregated into lighter fractions. Intact synaptic junctions that survived sonication appear to be retained at the interface above 1.2 M sucrose layer, as judged by the confluence of pre- and postsynaptic markers such as syntaxin and PSD-95 in that fraction (Fig. 2C). EM analysis of pellets shows a predominance of PSDs devoid of presynaptic attachment (Fig. 3), estimated to make up 96% of all PSD-containing structures.

Morphological and biochemical observations summarized above indicate that the designed protocol of sonication of the SPM fraction followed by density gradient centrifugation provides a rapid and convenient method for the preparation of a highly enriched PSD fraction. Further application of affinity-based methods, such as magnetic beads coated with antibodies to the PSD-specific protein PSD-95 [17] could yield PSDs of high purity.

We had previously reported that sonication of Triton-derived PSDs results in their fragmentation into sub-complexes [18]. In the present study, however, under an identical sonication protocol, no such fragments were observed in PSDs isolated without Triton treatment. Since the main difference between PSDs prepared by the Triton and sonication methods is the presence of an associated postsynaptic membrane in the latter, a role of the membrane in keeping PSD sub-complexes together is suggested.

In addition to providing a platform for the organization of PSD sub-complexes, the postsynaptic membrane is likely to affect specific folding and orientation of proteins on the cleft side of the PSD. Retention of the postsynaptic membrane may be crucial for the preservation of the native structure of membrane proteins such as neurotransmitter receptors, and for maintaining the specificity of their interactions with other proteins.

The above considerations altogether suggest that, compared to conventional Triton-derived PSDs, sonication-derived PSDs with attached postsynaptic membrane provide a better model for the elucidation of postsynaptic molecular organization. As it avoids detergent-induced alterations, the preparation is better suited for studies on protein-protein interactions within the PSD. For morphological studies, the presence of the membrane should allow easy orientation as cleft versus cytoplasmic surfaces and visualization of membrane-bound elements such as neurotransmitter receptors closer to their native state.

In conclusion, the demonstration of dissociation of the synaptic junction through sonication gives new insights on the nature of the association of pre- and postsynaptic compartments and provides a strategy for the isolation of PSDs retaining the postsynaptic membrane, a preparation that may prove to be a valuable for the elucidation of the molecular organization of the PSD.

## Electronic supplementary material

Below is the link to the electronic supplementary material.

Supplementary Material 1

## Data Availability

No datasets were generated or analysed during the current study.

## References

[1] Peters A, Palay SL, Webster HD. The fine structure of the nervous system. New York: Oxford University Pres; 1991.

[2] Cohen RS, Blomberg F, Berzins K, Siekevitz P. The structure of postsynaptic densities isolated from dog cerebral cortex. I. Overall morphology and protein composition. J Cell Biol. 1977;74:181–203. 194906 10.1083/jcb.74.1.181 PMC2109867

[3] Carlin RK, Grab DJ, Cohen RS, Siekevitz P. Isolation and characterization of postsynaptic densities from various brain regions: enrichment of different types of postsynaptic densities. J Cell Biol. 1980;86:831–45. 7410481 10.1083/jcb.86.3.831 PMC2110694

[4] Phillips GR, Huang JK, Wang Y, Tanaka H, Shapiro L, Zhang W, Shan WS, Arndt K, Frank M, Gordon RE, Gawinowicz MA, Zhao Y, Colman DR. The presynaptic particle web: ultrastructure, composition, dissolution, and reconstitution. Neuron. 2001;32:63–77. 11604139 10.1016/s0896-6273(01)00450-0

[5] Gordon-Weeks PR, Jones DH, Gray EG, Barron J. Trypsin separates synaptic junctions to reveal pre- and post-synaptic Concanavalin A receptors. Brain Res. 1981;219:224–30. 7260628 10.1016/0006-8993(81)90287-0

[6] Jordan BA, Fernholz BD, Neubert TA, Ziff EB. New tricks for an old dog: proteomics of the PSD. In: Kittler JT, Moss SJ, editors. The dynamic synapse: molecular methods in ionotropic receptor biology. Boca Raton (FL): CRC Press/Taylor & Francis; 2006. Chapter 3. 21204470

[7] Dosemeci A. Proteomic analysis of the postsynaptic density. In: Clelland J, editor. Advances in neurobiology 2, genomics, proteomics, and the nervous system. New York: Springer; 2011. pp. 227–49.

[8] Petersen JD, Chen X, Vinade L, Dosemeci A, Lisman JE, Reese TS. Distribution of postsynaptic density (PSD)-95 and Ca2+/calmodulin-dependent protein kinase II at the PSD. J Neurosci. 2003;23:11270–8. 14657186 10.1523/JNEUROSCI.23-35-11270.2003 PMC6741048

[9] Chen X, Vinade L, Leapman RD, Petersen JD, Nakagawa T, Phillips TM, Sheng M, Reese TS. Mass of the postsynaptic density and enumeration of three key molecules. Proc Natl Acad Sci U S A. 2005;102:11551–6. 16061821 10.1073/pnas.0505359102 PMC1182136

[10] DeGiorgis JA, Galbraith JA, Dosemeci A, Chen X, Reese TS. Distribution of the scaffolding proteins PSD-95, PSD-93, and SAP97 in isolated PSDs. Brain Cell Biol. 2006;35:239–50. 18392731 10.1007/s11068-007-9017-0

[11] Swulius MT, Kubota Y, Forest A, Waxham MN. Structure and composition of the postsynaptic density during development. J Comp Neurol. 2010;518:4243–60. 20878786 10.1002/cne.22451 PMC2948241

[12] Fera A, Dosemeci A, Sousa AA, Yang C, Leapman RD, Reese TS. Direct visualization of camkii at postsynaptic densities by electron microscopy tomography. J Comp Neurol. 2012;520:4218–25. 22627922 10.1002/cne.23151

[13] Jung JH, Chen X, Reese TS. Cryo-EM tomography and automatic segmentation delineate modular structures in the postsynaptic density. Front Synaptic Neurosci. 2023;15:1123564. 37091879 10.3389/fnsyn.2023.1123564 PMC10117989

[14] Tao-Cheng JH, Moreira SL, Winters CA, Reese TS, Dosemeci A. Modification of the synaptic cleft under excitatory conditions. Front Synaptic Neurosci. 2023;15:1239098. 37840571 10.3389/fnsyn.2023.1239098 PMC10568020

[15] Dosemeci A, Makusky AJ, Jankowska-Stephens E, Yang X, Slotta DJ, Markey SP. Composition of the synaptic PSD-95 complex. Mol Cell Proteom. 2007;6:1749–60. 10.1074/mcp.M700040-MCP200 PMC2096750 17623647

[16] Chapman ER, An S, Barton N, Jahn R. SNAP-25, a t-SNARE which binds to both syntaxin and synaptobrevin via domains that May form coiled coils. J Biol Chem. 1994;269:27427–32. 7961655

[17] Vinade L, Chang M, Schlief ML, Petersen JD, Reese TS, Tao-Cheng JH, Dosemeci A. Affinity purification of PSD-95-containing postsynaptic complexes. J Neurochem. 2003;87:1255–61. 14622105 10.1046/j.1471-4159.2003.02091.x

[18] Dosemeci A, Tao-Cheng JH, Bakly V, Reese TS. Postsynaptic densities fragment into subcomplexes upon sonication. Mol Brain. 2019;12:72. 31439005 10.1186/s13041-019-0491-y PMC6704671

