# Supplementary material for: Sonication dissociates the synaptic cleft and allows purification of postsynaptic densities with associated postsynaptic membrane
Source: Mol Brain. 2025 May 30;18:47. doi: 10.1186/s13041-025-01217-7 (PMC12123786; doi:10.1186/s13041-025-01217-7)
Supplement: Supplementary file 1 — Supplementary Material 1 [file 13041_2025_1217_MOESM1_ESM.docx]

**Supplementary Table 1: Fractionation of specific proteins upon sonication of SPM and sucrose density centrifugation**

|  | **Relative band intensity** | | | |
| --- | --- | --- | --- | --- |
| Protein | Supernatant | Interphase above 0.85 M sucrose layer | Interphase above 1.2 M sucrose layer | Pellet  (PSD prep) |
| PSD-95 | 0 | 0 | 0.46 | 1 |
| SynGAP | 0 | 0 | 0.41 | 1 |
| Syntaxin | 0.58 | 3.26 | 3.49 | 1 |

Relative band intensities corresponding to specific proteins were estimated from Western immunoblots, using BioRad Image Lab Software. The intensity at the PSD-enriched pellet was set as 1.
